# Symmetry-Projected Nuclear-Electronic Hartree–Fock: Eliminating Rotational Energy Contamination

**DOI:** 10.1021/acs.jpca.3c04822

**Published:** 2023-10-13

**Authors:** Robin Feldmann, Alberto Baiardi, Markus Reiher

**Affiliations:** ETH Zürich, Department of Chemistry and Applied Biosciences, Vladimir-Prelog-Weg 2, Zürich 8093, Switzerland

## Abstract

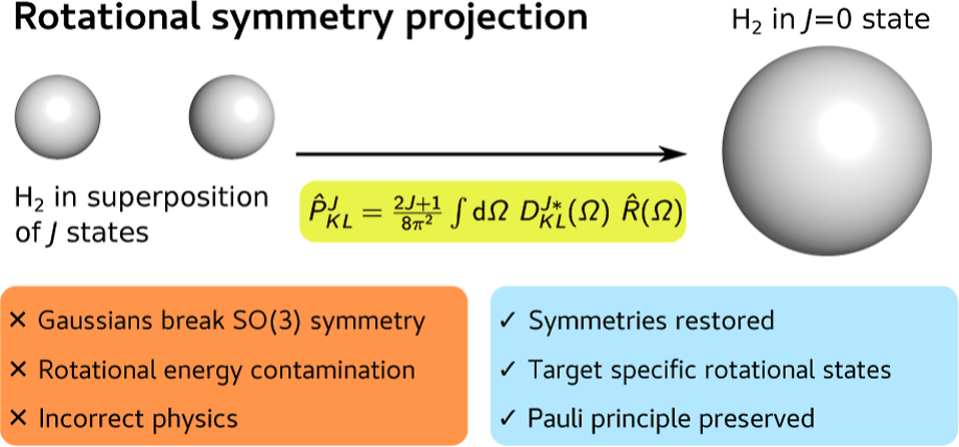

We present a symmetry projection technique for enforcing rotational and parity symmetries in nuclear-electronic Hartree–Fock wave functions, which treat electrons and nuclei on equal footing. The molecular Hamiltonian obeys rotational and parity inversion symmetries, which are, however, broken by expanding in Gaussian basis sets that are fixed in space. We generate a trial wave function with the correct symmetry properties by projecting the wave function onto representations of the three-dimensional rotation group, i.e., the special orthogonal group in three dimensions SO(3). As a consequence, the wave function becomes an eigenfunction of the angular momentum operator which (i) eliminates the contamination of the ground-state wave function by highly excited rotational states arising from the broken rotational symmetry and (ii) enables the targeting of specific rotational states of the molecule. We demonstrate the efficiency of the symmetry projection technique by calculating the energies of the low-lying rotational states of the H_2_ and H_3_^+^ molecules.

## 1 Introduction

Solving the molecular Schrödinger equation by considering both nuclei and electrons on equal footing^1^ incorporates effects beyond the Born–Oppenheimer approximation in molecular simulations.^2^ These effects are the result of the coupling between nuclear and electronic degrees of freedom, i.e., nonadiabatic and nuclear quantum effects. Hence, phenomena which are governed by these effects benefit from such pre-Born–Oppenheimer methods.

Formally, pre-Born–Oppenheimer theories harbor the advantage over Born–Oppenheimer-based methods that the nonrelativistic interaction potential between nuclei and electrons is given exactly by pairwise Coulomb interactions. By contrast, in the case of the latter methods, the potential entering the vibrational Hamiltonian is an *N*-body potential that must be approximated with fitting or interpolation algorithms.^3,4^ This also implies that the coupling between nuclear degrees of freedom of different kinds (e.g., rovibronic coupling) is treated directly in pre-Born–Oppenheimer theories. Moreover, also the nuclear permutation and spin symmetries are considered from the outset.

Both the nuclei and the electrons can be described with single-particle functions, which are referred to as orbitals. Thomas pioneered this approach in 1969,^5−8^ and Petitt proposed the first nuclear electronic Hartree–Fock method^9^ which was developed into a practical method by Nakai and co-workers.^10^ Since then, many electronic structure methods were extended to treat nuclei quantum mechanically: including Møller–Plesset perturbation theory,^11−14^ configuration interaction,^15−19^ density matrix renormalization group,^20,21^ coupled cluster,^22−27^ and density functional theory.^28−31^

The molecular Hamiltonian possesses continuous symmetries. Translational and rotational symmetries must therefore be respected by any wave function ansatz. Solving the molecular Schrödinger equation based on atom-centered single-particle functions, however, breaks the continuous symmetries of the Hamiltonian. A consequence of this symmetry breaking is that variational methods yield inaccurate energies even for very flexible and fully optimized wave functions.^20,32^ Although the translational energy can be subtracted exactly from a nonrelativistic Hamiltonian, breaking the symmetry can introduce spurious excited states in its spectrum.^15^

Nakai et al. showed that the problem of rotational energy contamination can be remedied by approximately eliminating the rotational energy from the Hamiltonian.^32−34^ In their approach, the rigid-body rotational energy is expanded around an equilibrium geometry in a Taylor series which was shown to converge below 1 mHa already at zeroth order.^33^ However, this approach is limited solely to rotational ground states. Moreover, it effectively changes the Hamiltonian from the exact molecular Hamiltonian to an approximate one in which all nuclei are distinguishable.

Heller and Blanco introduced the projection onto rotational states within the Born–Oppenheimer approximation.^35^ Later, we extended this approach by projecting pre-Born–Oppenheimer wave functions expressed in terms of explicitly correlated Gaussians onto states exhibiting proper rotational symmetry.^36^

In this work, we introduce a method to eliminate exactly the rotational energy contamination to the nuclear-electronic Hartree–Fock (NE-HF) wave function by enforcing the correct rotational symmetry of the wave function through symmetry projection. The projection is carried out in the projection-after-variation fashion, i.e., the orbitals are optimized by standard nuclear-electronic Hartree–Fock calculations, and the wave function is subsequently symmetry projected. This method represents a first step toward accurately calculating the vibrational and rotational spectra of small to moderately sized molecules without the Born–Oppenheimer approximation. We adopt the approach from nuclear structure theory^37−40^ and present its theoretical foundations in Section 2. Then, Section 3 provides the necessary details for its implementation. After a description of computational details in Section 4, we apply our approach to calculate the energies of rotational states of H_2_ and H_3_^+^ in Section 5. We selected H_2_ and H_3_^+^ for scrutinizing our approach since the symmetry properties of these molecules are well understood, and highly accurate reference data are available in the literature.

## 2 Theory

### 2.1 Symmetry Properties of the Molecular Schrödinger Equation

The translation-free molecular Hamiltonian for a system comprising *N*_p_ particles with masses *m*_*i*_, charges *q*_*i*_, and positions  is given as^20^
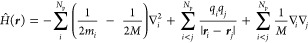
1where  collects the positions of all particles,  is the total mass, and  is the derivative operator with respect to the position of particle *i*. While the Born–Oppenheimer electronic Hamiltonian treats nuclear coordinates as fixed parameters, the molecular Hamiltonian treats the nuclear positions explicitly as quantum mechanical operators. As a result, the molecular Hamiltonian is of higher symmetry than the Born–Oppenheimer electronic Hamiltonian. Specifically, the Born–Oppenheimer Hamiltonian obeys a discrete point group symmetry that originates from the fixed nuclear positions, whereas the molecular Hamiltonian has continuous translational and rotational symmetry, as well as discrete spatial inversion symmetry. Translational symmetry specifies that the system remains unchanged under a constant shift, ***a***
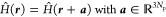
2

Equation 1 fulfills eq 2 because the derivative operator is invariant upon overall translations and the Coulomb interaction depends only on the relative positions of the particles, which remain unchanged upon translation. Rotational symmetry demands that the system remains unchanged under a rotation
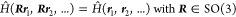
3here, SO(3) is the group of orthogonal three-by-three matrices with determinant 1, defined as
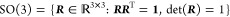
4

Both the derivative operator and the Coulomb interaction are isotropic, and, therefore, they are invariant upon symmetry transformations of the SO(3) group. The group of spatial inversions, *C*_I_ = {**1**, ***I***}, consists of only two elements: the identity **1** and the inversion matrix ***I***, which is defined as ***Ir*** = −***r***. It is easy to prove that the Hamiltonian is invariant also upon spatial inversion, that is
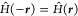
5

The symmetries of the Hamiltonian dictate that the wave function must transform according to irreducible representations of the corresponding symmetry groups. As a result, exact solutions to the Schrödinger equation can be labeled according to these irreducible representations, which serve as good quantum numbers. The ground-state translational energy has already been removed in the Hamiltonian of eq 1, and therefore, we omit the corresponding symmetry label. The irreducible representations of the SO(3) group are labeled by the angular momentum quantum number *J* = 0, 1, ... and its projection onto the *z*-axis, denoted by *M*_*J*_ = −*J*, – *J* + 1, ..., *J*. Additionally, we denote the parity quantum number as *p* = ±1 for even and odd parity, respectively.

An additional wave function symmetry is given by the spin, which is represented by the SU(2) group with the spin quantum numbers for particles of type *I* given as  and *M_S,I_* = −*S_I_*, −*S_I_* + 1, ..., *S_I_*. In our approach, we rely on the unrestricted nuclear-electronic Hartree–Fock ansatz, which only allows us to specify *M_S,I_*, as Slater determinants (or permanents) are generally not eigenfunctions of the  operator. The wave function ansatz reads
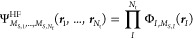
6with the number of particle types, *N*_t_, and ***r****_I_*, the vector that contains the coordinates of all particles of type *I*.  is constructed as a properly symmetrized product of nuclear or electronic molecular orbitals, ϕ*_Isi_*
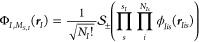
7where *s_I_* is the single-particle spin quantum number, while ***r***_*Iis*_ labels the coordinate of the *i*th particle of type *I* with spin-projection *s*. *N*_*Is*_ denotes the number of particles of type *I* with spin projection on the *z*-axis *s*. The index *s* runs over all possible values of the magnetic quantum number *m*_*s*,*I*_. We note that *M_*S*,I_* is fixed by the specified number of particles of type *I* for each spin *s*. The (anti)symmetrization operator, , enforces the correct permutational symmetry of the product of orbitals, i.e., by antisymmetrization for fermions and by symmetrization for bosons.

The molecular orbitals of particle type *I* are constructed as a linear combination of *L_I_* Gaussian-type orbitals (GTOs), *G*_*I*μ_, with pre-optimized Gaussian widths, contraction coefficients, and shifts as
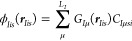
8where we restrict ourselves to solid harmonic contracted GTOs. The expansion into GTOs, however, comes with the severe drawback that the wave function ansatz breaks the rotational, translational, and parity symmetries of the Hamiltonian. That is, it does not transform according to irreducible representations of these symmetry groups. In fact, the centers of the GTOs are located at fixed points in space—usually at the position of the nuclei predicted based on a Born–Oppenheimer calculation. Since Gaussian basis sets have anisotropic distributions when not centered at the center of mass of the molecule, they inherently break the rotational symmetry.

### 2.2 Restoring Wave Function Symmetries by Symmetry Projection

Since the use of GTOs breaks the rotational symmetry, the exact expansion of the Hartree–Fock wave function is given as a linear combination of eigenstates of the Hamiltonian with various *J* states. This symmetry-breaking results in unreasonably high energies,^32^ even for fully variationally optimized wave functions, since states with arbitrarily high *J* values can contribute significantly to the wave function. We address this issue by introducing a method to project out all *J* states in the Hartree–Fock wave function except the one of interest. For simplicity, we focus on the rotational symmetry.

We denote the Hilbert space in which the exact solution to the molecular Schrödinger equation is defined as . A complete basis for  can be constructed from the eigenstates  of *H^*, with *n* representing all quantum numbers except the rotational ones such that
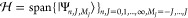
9

As a result,  decomposes into a series of invariant subspaces associated with the irreducible representations of SO(3) and the remaining quantum numbers *n* as
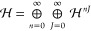
10where the subspaces, , are at least 2*J* + 1 dimensional depending on other symmetries (contained in *n*) or accidental degeneracies.

The Hartree–Fock wave function, |Ψ^HF^⟩, can be expanded in the basis  as
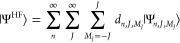
11

We consider eq 11 which is easily generalized to a general symmetry-broken wave function as a linear combination of functions that transform according to irreducible representations of the rotational symmetry group. A projection operator, , can remove the contributions from all states that are not associated with a specific set of *J* and *M*_*J*_ values. The resulting wave function, , becomes an eigenfunction of the total angular momentum operator  with quantum numbers *J* and *M*_*J*_. However, depending on the orientation of the symmetry broken state |Ψ^HF^⟩, the composition of eq 11 varies, and it may be that the projection operator projects onto the null vector if  for all *n*. To overcome this issue, we consider the projection on all possible *M*_*J*_ for a given *J*. In Dirac’s bra-ket notation, the projection operator can be written as
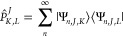
12from which the following key properties follow
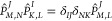
13
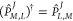
14 is an orthogonal projection operator in the strict mathematical sense, i.e., it satisfies the properties , only for *K* = *L*. When *K* ≠ *L*, the operator is sometimes referred to as the “shift operator” or “transfer operator”.^40^ We now apply the operator to the Hartree–Fock wave function in eq 11 and obtain
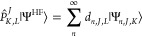
15

To remove the dependence on the orientation of |Ψ^HF^⟩ and the specified *M*_*J*_ value in the ansatz, we need to diagonalize the Hamiltonian in the subspace spanned by the projected wave functions  for all *K* and *L*. To that end, we first note that the Hamiltonian and overlap matrices expressed in the  basis are block-diagonal, with one block per *J* value^40^
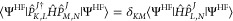
16
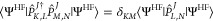
17where we employed  and the properties in eqs 13 and 14. The above equations provide a way to define the Hamiltonian and overlap matrices within a given angular momentum-irreducible representation of dimension 2*J* + 1, defined as follows
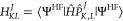
18
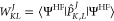
19

Diagonalization of the Hamiltonian in this subspace yields the projected Hartree–Fock wave function as
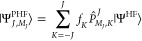
20which is an eigenfunction of  and is independent on the orientation of the symmetry-broken state |Ψ^HF^⟩. The coefficients *f*_*K*_ can be determined by solving the Hill–Wheeler equations^37,40^
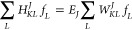
21and the energies are the generalized eigenvalues, *E*_*J*_. By comparing eqs 11 and 15, we note that via the symmetry projection, we can eliminate the contribution of higher lying rotational states. For this reason, the symmetry-projected energy is a better variational estimate of the exact one. We now proceed by deriving the explicit expression for the projection operator.

### 2.3 Angular Momentum and Parity Projection Operators

A rotation in a three-dimensional space can be uniquely represented by three consecutive rotations around the Euler angles, denoted as Ω = (α, β, γ). In this work, we adopt the convention of active rotations^41^ where the three rotations are performed in the following order: (1) rotate the system by an angle 0 ≤ γ ≤ 2π about the *z*-axis, (2) rotate the system by an angle 0 ≤ β ≤ π about the *y*-axis, and (3) rotate the system by an angle 0 ≤ α ≤ 2π about the *z*-axis. With this convention, we can write down a generic rotation ***R*** ∈ SO(3) as a product of rotation matrices according to^41^
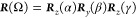
22where the rotation matrices read
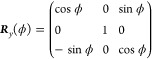
23and
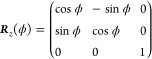
24

We introduce the generator of rotations around an axis *i* ∈ {*x*, *y*, *z*}, denoted by ***J***_*i*_, as the derivative of the rotation matrices, ***R***_*i*_ ∈ SO(3), according to
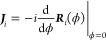
25

The ***J***_*i*_ operators are the 3 × 3 matrix representations of the well-known angular momentum operators (in Hartree atomic units). Any three-dimensional rotation matrix can be parametrized in terms of the generators as
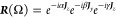
26

Since the action of ***J***_*i*_ is only defined on , we introduce the representations of the generators of rotation for a generic Hilbert space as  and we write the general representation of the rotation operator as
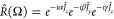
27where *R̂*(Ω) and  act on many-particle and single-particle Hilbert spaces. The action of the rotation operator of eq 27 on an arbitrary angular momentum eigenstate |*J*, *M*_*J*_⟩ can be expressed with the closure of the angular momentum basis as
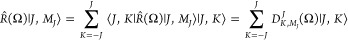
28where the expansion coefficients are given by the Wigner D-matrices, .^41^ With this, we can derive the explicit expression of the action of the angular momentum projection operator onto the symmetry-broken Hartree–Fock wave function. To that end, we write down the action of the rotation operator from eq 27 on the symmetry-broken Hartree–Fock wave function from eq 11, which was expanded in the eigenstates of the Hamiltonian as
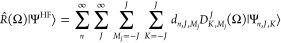
29where we expanded a rotated angular momentum state as a sum of eigenstates with the same *J*. We exploit the orthogonality of the Wigner D-matrices, i.e.
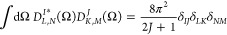
30with
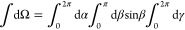
31

We multiply eq 29 by *D*_*L*,*N*_^J*^(Ω) and integrate over Ω, which yields
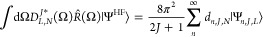
32after exploiting eq 30. The right-hand side of eq 32 is equivalent to eq 15 up to the constant pre-factor. Hence, we can identify the projection operator as
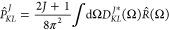
33

The energy of the projected wave function can be calculated by solving the Hill–Wheeler equation, eq 21, for the Hamiltonian operator
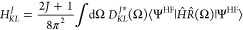
34and the overlap
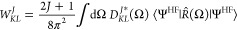
35where the integration in the matrix element and the overlap of the Hartree–Fock wave function are carried out over the electronic and nuclear coordinates. To project onto states with a given parity, we define the parity projection operator as^37^
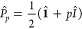
36which can be applied to the Hartree–Fock wave function as
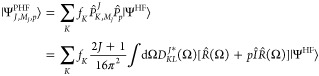
37

Hence, the matrix elements to be evaluated are
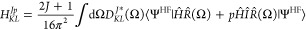
38
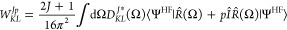
39

These previous are the key to our symmetry-projected NE-HF method.

## 3 Implementation of the Projected NE-HF Method

### 3.1 Discretization of the Projection Operator

We discretize the integrals entering the Hill–Wheeler equations, and since the parity projection is straightforward as opposed to the rotational projection, we disregard the former and write the projected matrix elements as
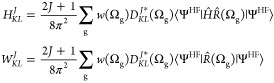
40where the weights *w*(Ω_*g*_) are determined by the quadrature methods chosen.^42,43^ The most efficient scheme is either a Lebedev quadrature for the angles α and β, and a periodic trapezoidal quadrature for the γ angle (denoted as Lebedev trapezoidal), or a periodic trapezoidal quadrature for α and γ, and a Gauss quadrature for cos β (denoted as Gauss trapezoidal). For a detailed recent study on quadrature rules for angular momentum projection, see ref (43). The numerical integration can be easily parallelized over the quadrature points since the matrix elements at each quadrature point, *g*, can be evaluated independently. The final step for evaluating the matrix elements requires us to investigate how the rotation operator acts on the Hartree–Fock wave function and to evaluate the nonorthogonal matrix elements. We will address these points in the next section.
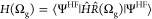
41
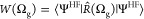
42

### 3.2 Rotation of Basis Functions

The Hartree–Fock wave function is composed of orbitals that are defined in terms of GTOs. Hence, rotating the wave function requires evaluating the action of the rotation operator on a GTO. For readability, we omit the particle-type index *I* in the following. We rely on real-valued solid harmonic GTOs, which are (up to the normalization factor) given as^44^
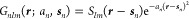
43where *a*_*n*_ is the Gaussian width, ***s***_*n*_ is the shift, and  is the real-valued solid harmonic, as defined, e.g., in ref (44). *n* is the shell index, while  and *m* are the angular momentum quantum numbers of the solid harmonics. The rotation of the GTO reads
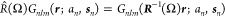
44

The rotation of the Gaussian function (i.e., the second term of the right-hand side of eq 43) is straightforward to evaluate
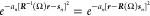
45since ***R***^–1^(Ω) = ***R***^T^(Ω). Therefore, the rotated Gaussian is equivalent to the original Gaussian with a rotated center. We rotate the solid harmonics as described in ref (45). We exploit the matrix representation of a rotation in the solid harmonic basis ***X***(Ω) as defined in ref (45) to express the rotation of the GTO as
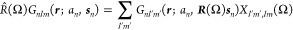
46

Next, we write a matrix element for an arbitrary one-body operator, *O^*, in the GTO basis with the center of the Gaussian in the ket rotated as
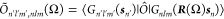
47where we omitted the width of the Gaussian for readability. We now obtain the matrix element where both the center of the Gaussian and the solid harmonics are rotated according to the ***X*** matrix as follows
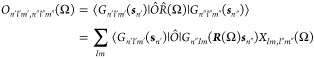
48

We can rewrite this equation compactly in matrix notation, where ***X***(Ω) is extended to the whole GTO space as  such that
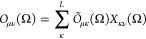
49where the Greek indices are introduced as combined indices as μ = (*n*, , *m*). Consequently, for the rotation of a Hartree–Fock wave function, we first calculate the integrals in the GTO basis where the Gaussian shifts of the GTOs in the ket are rotated by Ω. Then, we evaluate the rotation matrix ***X***(Ω) and apply it to the integrals. Two-body integrals can be rotated in complete analogy by rotating both Gaussian functions in the ket according to
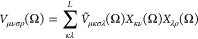
50where  is a two-body integral in the chemistry notation over (possibly contracted) GTOs, χ_μ_(***r***), where the centers in the ket are rotated
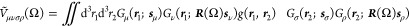
51

For the parity projection, *I^* is applied to GTOs by inverting the origin of the GTO in such a way that ***s***_*n*_ → −***s***_*n*_ and the solid harmonics transform under the parity inversion as . This operation can be easily incorporated into ***X***(Ω) by multiplying the diagonal with “–1” if  is odd.

### 3.3 Matrix Elements and Expectation Values

We evaluate the matrix elements following the approach of ref (46). Note that the matrix elements cannot be evaluated with the strategy proposed in refs (47) and (42) for spin projection because a truncated GTO expansion does not constitute a complete basis in . In fact, a GTO rotated by an arbitrary angle Ω cannot be represented, in general, in a finite linear combination of GTOs with a given fixed center. We define the rotated GTO as
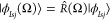
52and the overlap matrix *B*_*Isij*_(Ω) as
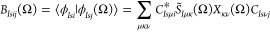
53 is the overlap matrix in the GTO basis with the Gaussians in each ket rotated by Ω. The *ij* cofactor of ***B***_*Is*_ is defined as
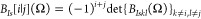
54

We evaluate the matrix elements of the one-body contribution, *H*_1,*I*_, for particles of type *I* according to the Löwdin rules^20,48^ as
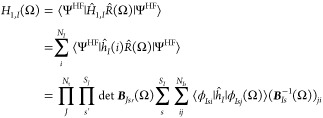
55where we have employed Cramer’s rule
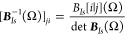
56

Following the approach of Thom and Head-Gordon,^46^ we evaluate eq 55 in the Löwdin-paired basis
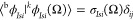
57where ⟨^*b*^ϕ_*Isi*_| are the bra orbitals and |^*k*^ϕ_*IsJ*_(Ω)⟩ the ket orbitals. This basis can be derived by a singular value decomposition of the overlap matrix
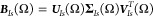
58where **Σ**_*Is*_(Ω) = diag(σ_*Is*1_(Ω), ...) collects the singular values, while ***U***_*Is*_(Ω) and ***V***_*Is*_(Ω) denote the unitary transformation matrices defining the bra and rotated ket orbitals, respectively. Note that the diagonalization of the inverse overlap matrix is obtained with the same transformation matrices as
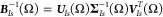
59which gives the inverse singular values. ***U***_*Is*_ defines a transformation that can be applied to the bra orbital coefficients as
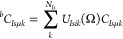
60and, similarly, ***V***_*Is*_ gives a transformation of the ket orbital coefficients as
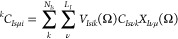
61where ***X***_*I*_(Ω) = {*X*_*I*μν_(Ω)} is the solid harmonics rotation matrix introduced in eq 49. Note that in order to be a phase-conserving orbital rotation, we exploit the fact that det(***U***_*Is*_(Ω)) = det(***V***_*Is*_(Ω)) = 1, which can be enforced by dividing the matrices by the value of their determinant. Including the solid harmonics rotation matrix in the definition of the molecular orbital coefficients is advantageous as we do not need to first rotate the orbitals in the GTO basis and then calculate an expectation value, instead both can be achieved in a single step. This is equivalent to including the orthonormalization matrix of the GTO overlap in the definition of the molecular orbitals which is the standard in the Hartree–Fock method. Additionally, to write the matrix elements more compactly, we define
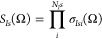
62
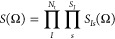
63By inserting **1** = ***U***_*Is*_^T^(Ω)***U***_*Is*_(Ω) and **1** = ***V***_*Is*_^T^(Ω)***V***_*Is*_(Ω) in eq 55 allows us to simplify the matrix element as^46^
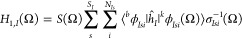
64where we employed
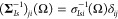
65

To formulate the expectation value in the GTO basis, we introduce the codensity matrix as
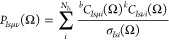
66which allows us to evaluate the matrix elements with the same procedure as for an orthonormal basis
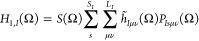
67where  is the GTO representation of the operator where the GTO centers are rotated in the ket. The two-body matrix elements can be derived based on our previous works,^20,21,49^ which we generalize here to a Löwdin-paired basis. The two-body matrix elements for operators coupling particles of different types can again be evaluated with Cramer’s rule. For the evaluation of matrix elements of operators coupling particles of the same type, we introduce the *ik*, *jl* cofactor as
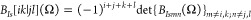
68which can be rewritten with the generalization of Cramer’s rule^48^
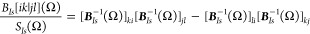
69

To further evaluate all two-body matrix elements, we insert the identity in terms of the transformation matrices for both particles in analogy to eq 64 and insert the definition of the codensity matrix of eq 66. Consequently, we can write the Hamiltonian matrix element, eq 41, at a quadrature point as
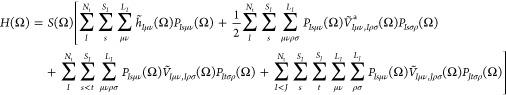
70

Here,  denotes the one-body matrix elements in the GTO basis with the center of the GTO rotated in the ket, as in eq 47, and  refers to the two-body matrix element where the centers of both GTOs in the ket are rotated, as in eq 51. The superscript in  indicates that the integral is antisymmetrized. The overlap matrix element, eq 42, is given by *W*(Ω) = *S*(Ω). Note that the computational scaling of evaluating the expectation value of the Hamiltonian in the Löwdin-paired basis can be reduced from  to . The former scaling stems from the fact that the two-body integrals have to be transformed to the molecular orbital basis with the integral direct method. Conversely, in the Löwdin-paired basis, the contraction of the two-body integrals with the codensity can be carried out equivalently to the unprojected Hartree–Fock theory.^50^ Consequently, the computational scaling at each quadrature point is the same as in unprojected NE-HF.^21,49^ At each quadrature point, we evaluate eq 70 with an integral-direct method. Finally, we solve the Hill–Wheeler equations that read, in matrix form,
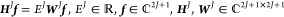
71where ***H***^*J*^ and ***W***^*J*^ are Hermitian and ***W***^*J*^ can have eigenvalues λ_*k*_ ≥ 0. As a consequence, ***H***^*J*^ has only as many eigenvalues as ***W***^*J*^ has nonzero eigenvalues. We solve the generalized eigenvalue problem in eq 71 with the canonical Löwdin orthonormalization method (see, e.g., ref (20)) that is also commonly used to solve the Roothaan–Hall equations: we first diagonalize ***W***^*J*^ and discard all eigenvalues and their eigenvectors that are below a given numerical threshold. Then, assuming that there are *k* nonzero eigenvalues, we set up the transformation matrix  that orthonormalizes the overlap as ***Y***^T^***W***^*J*^***Y*** = **1**_*k*×*k*_, such that we can transform the Hill–Wheeler equations into a Hermitian standard eigenvalue problem
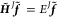
72where .

### 3.4 Spin Expectation Value

We conclude the implementation section by deriving the expectation value of the  operator
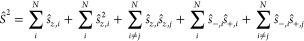
73which can be calculated efficiently within the Löwdin-paired basis. For clarity, we focus here on a single-particle-type wave function with spin orbitals and evaluate the matrix elements between |Φ⟩ with basis functions {|ϕ_*i*_⟩} and |Ψ⟩ with basis functions {|ψ_*i*_⟩}, and the overlap is *B*_*ij*_ = ⟨ϕ_*i*_|ψ_*j*_⟩. We first introduce the spin-specific overlap
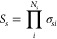
74and the reduced overlap vector *a*_*i*_^*s*^
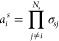
75with those, we can express the expectation value of the first term in eq 73 as
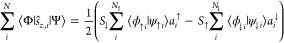
76

For the second term, we have
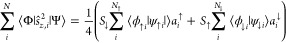
77

In contrast to the other terms, the expectation value of the third term is evaluated in the spin–orbital basis and is given by
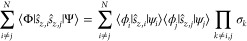
78

The fourth term can be evaluated according to
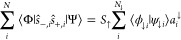
79and, finally, the expectation value of the final term is expressed as
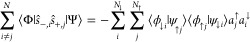
80which completes the evaluation of the expectation value of the  operator within the Löwdin-paired basis.

## 4 Computational Details

For this work, we employed the correlation-consistent (cc) basis sets^51^ for electrons, augmented with specialized functions for multicomponent (mc) calculations developed by Brorsen et al.^52^ For the protons, we chose the protonic basis (PB) by Hammes-Schiffer and co-workers.^53^ We implemented the projected NE-HF method in the open-source Kiwi program.^54^ We calculated the integrals with our integral evaluation package^55^ which relies on Libint.^56^ For H_2_, we set the bond length to 0.74 Å, while for H_3_^+^, we adopt an equilateral triangular structure with an edge length of 0.867850 Å. Moreover, we calculated the classical center of mass of the system based on the centers of the Gaussians, and we transform the coordinates such that the center of mass is in the origin of the coordinate system. For H_2_, the center of mass is the center of the bond, and for H_3_^+^, it is the center of the equilateral triangle. Note that the overlap matrix of eq 53 may be singular. However, a singular overlap matrix indicates that the corresponding quadrature point yields a null-energy contribution. We, therefore, neglected quadrature points for which the overlap matrix contains singular values with an absolute value smaller than a numerical threshold of 10^–5^ in atomic units. The matrix elements *H*(Ω_g_) and *W*(Ω_g_) can be evaluated independently for different quadrature points, *g*. We, therefore, parallelized the construction of the ***H***^*J*^ and ***W***^*J*^ matrices via shared-memory OpenMP parallelization. Specifically, each thread evaluates *H*(Ω_g_) and *W*(Ω_g_) for a subset of quadrature points and accumulates the result into thread-specific local ***H***^*J*^ and ***W***^*J*^ matrices. The local matrices of each thread are then added to construct the full Hill–Wheeler matrices.

## 5 Results and Discussion

### 5.1 Convergence of the Quadrature

We first investigated the convergence of the symmetry-projected NE-HF energy with the number of quadrature points of the numerical integration for the H_2_ molecule in the *J* = 0 and  state. As we mentioned above, the action of the projection operator on the Hartree–Fock wave function depends on the orientation of the symmetry-broken Hartree–Fock wave function. Therefore, the numerical integration can be more or less efficient depending on the positions of the centers of the GTOs. Not surprisingly for a linear molecule, we found that orienting the basis functions along the *z*-axis leads to the fastest energy convergence. Moreover, in this case, the combination of trapezoidal and Gauss quadrature converged faster than the Lebedev trapezoidal quadrature. The convergence of the ground-state energy with the number of quadrature points per angle is shown in Table 1. The ground-state energy was converged at least up to 1 μHa with *N*_*q*_ = 20 points per angle, yielding *N*_*q*_^3^ = 8000 quadrature points in total.

**Table 1:** Convergence of the Energy in Atomic Units with the Number of Quadrature Points per Angle, *N*_*q*_, and the Total Number of Quadrature Points, *N*_*q*_^3^, of the Gauss-Trapezoidal Quadrature for the H_2_ System^a^

*N*_*q*_	*N*_*q*_^3^	*cc*-pVDZ-mc	*cc*-pVTZ-mc	*cc*-pVQZ-mc
10	1000	–1.096932	–1.097670	–1.097793
15	3375	–1.095345	–1.096069	–1.096171
20	8000	–1.095341	–1.096061	–1.096165
25	15,625	–1.095341	–1.096061	–1.096165
30	27,000	–1.095341	–1.096061	–1.096165

a We fix the proton basis set to PB4-D and vary the electronic basis set.

### 5.2 Absolute and Relative Energies of H_2_

We investigated the convergence of the absolute energies and energy differences between the  and  states given in Table 2. The absolute energy converges with increasing electronic basis set size, but no convergence is observed with the protonic basis set size. We already discussed this effect in our previous work^21^ where we ascribed the lack of convergence to the fact that the PB basis sets were optimized only for correlated calculations. Table 2 also contains that the energy differences with different combinations of basis sets are in the range of 2 cm^–1^, and hence, we may conclude that the energy difference is converged already with the smallest basis set combination.

**Table 2:** Convergence of the Energy with the Electronic (*cc*-pVDZ-mc, *cc*-pVTZ-mc, and *cc*-pVQZ-mc) and Protonic (PB4-D, PB4-F1, and PB5-G) Basis Set for the *J* = 0 and *J* = 1 States and for the Transition Energy Δ*E*_1←0_ of H_2_^a^

p^+^/e^–^	*cc*-pVDZ-mc	*cc*-pVTZ-mc	*cc*-pVQZ-mc
*E*_HF_			
PB4-D	–1.073789	–1.074474	–1.074572
PB4-F1	–1.073752	–1.074437	–1.074534
PB5-G	–1.073842	–1.074525	–1.074626
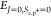			
PB4-D	–1.095341	–1.096061	–1.096139
PB4-F1	–1.095273	–1.095990	–1.096068
PB5-G	–1.095436	–1.096153	–1.096123*
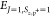			
PB4-D	–1.094685	–1.095406	–1.095485
PB4-F1	–1.094617	–1.095337	–1.095412
PB5-G	–1.094789	–1.095495	–1.095577*
Δ*E*_1←0_/cm^–1^			
PB4-D	144.12	143.91	143.66
PB4-F1	144.00	143.48	144.04
PB5-G	142.18	144.42	142.96*

a Absolute energies are given in atomic units and relative energies in cm^–1^. We chose the Gauss-trapezoidal quadrature with 20 points for each angle, resulting in 8000 quadrature points in total. *25 quadrature points for each angle and 15,625 points overall were required to reach convergence for the largest basis set combination of *cc*-pVQZ-mc and PB5-G.

Next, we compare the energy difference between rotational states to the value calculated by Pachucki and Komasa,^55^ which was obtained based on the Born–Oppenheimer approximation where the potential energy surface was generated with a wave function composed of explicitly correlated Gaussians, and the one-dimensional nuclear equation was solved numerically on a grid. Compared to their result of 118.55 cm^–1^, we find a deviation of 24.41 cm^–1^ to our energy difference of 142.96 cm^–1^. We note that since the nuclear Schrödinger equation arising from the Born–Oppenheimer approximation was solved numerically on a grid in the reference work, no projection onto rotational states was necessary.

Given the well-known limitations of the NE-HF method, as previously discussed in the literature,^1,21^ the observed deviation is likely due to the missing correlation energy. Another contributing factor could be that our method optimizes first the NE-HF wave function and then projects it onto the rotational state of interest (known as projection-after-variation) without accounting for orbital relaxation. This effect is included in a variation-after-projection approach that is computationally more demanding, and it requires the derivation of gradients of the projected energy with respect to the orbital coefficients or orbital rotations.^37^

An alternative approach for removing rotational contributions from NE-HF has been proposed by Nakai et al.^32^ Within their approach, an approximate correction term, based on expanding the rigid-rotor rotational energy around an equilibrium geometry, is included to remove rotational energy contribution from the Hamiltonian. The equilibrium geometry, however, is introduced a posteriori, and it breaks the nuclear indistinguishability. The resulting Hartree–Fock energy without rotational energy is lower by 29.774 mHa compared to the energy including the rotational contribution. With our method, we observe a lowering of the energy by 21.497 mHa. The deviation to Nakai’s result is 8.277 mHa, which was obtained with a different basis set.

We emphasize that the value of 21.497 mHa is equivalent to 4718.05 cm^–1^. Considering the rotational constant of 59.275 cm^–1^, this finding indicates that very high rotational states contribute to the symmetry-broken wave function. Moreover, Nakai’s approach includes orbital relaxation effects because it removes rotational contributions from the Hamiltonian that is used in the variational minimization. This suggests that the remaining energy lowering may be due to the missing orbital relaxation effects which could be included with a variation-after-projection scheme. However, while the approach of ref (32) can target only the rotational ground state, our algorithm can target rotational states with arbitrary *J* values. The possibility of targeting rotationally excited states is a key advantage that makes our method applicable to high-resolution spectroscopy calculations. This will allow for the computation of rotational spectra which is not possible when the rotational motion of the system is eliminated from the Hamiltonian.

A second advantage is the possibility of fully controlling the rotational symmetry properties of the wave function, which allows us to capture essential physics. For instance, in the H_2_ molecule, the *J* = 0 state is Pauli-forbidden for . If we attempt to project onto this state in our calculations, we will observe that it is forbidden since the ***W***^*J*^ matrix is numerically zero, i.e., the projector projects onto the null vector. The same holds true for the *J* = 1 state, which is forbidden for . Additionally, the NE-HF wave function for  exhibits broken symmetry in the nuclear spin. By implementing the spin expectation value for the projected NE-HF wave function, we observed that in this case, spin symmetry is exactly restored as a byproduct of the symmetry projection. The spin expectation values were evaluated according to the equations provided in Section 3.4.

Last, we have calculated the overlap of the symmetry-broken Hartree–Fock wave function with the projected Hartree–Fock wave function for the ground state with the *cc*-pVTZ-mc and PB4-D basis sets. The numerical evaluation yielded a value of ⟨Ψ_HF_|Ψ_0,0,1_^PHF^⟩ = 0.087839. Hence, the symmetry-broken wave function barely overlaps with the projected wave function. The fact that a Hartree–Fock wave function is considered qualitatively inaccurate in electronic structure theory when the overlap with the full-CI wave function (which corresponds to the *C*_0_ coefficient) is less than, say, 0.9 signifies that the symmetry-broken nuclear-electronic wave function does not resemble the correct wave function at all.

### 5.3 Rotational States of H_3_^+^

In this section, we investigate the absolute and relative energies of the H_3_^+^ molecule where parity must also be considered. Based on our findings from Section 5.2, which show no significant effect on relative energies for the chosen basis set combinations, we selected the combination of the PB4-D and *cc*-pVTZ-mc basis sets for all calculations on H_3_^+^. We centered the Gaussian functions of one hydrogen atom on the *z*-axis. We analyzed the energy convergence with the quadrature method and found that the combination of the Lebedev and trapezoidal rule yields faster convergence than the Gauss-trapezoidal quadrature. The quadrature is fully converged with 30 quadrature points for the trapezoidal rule and with 434 quadrature points for the Lebedev rule, resulting in 13,020 points in total.

The *J* = 0 state of the H_3_^+^ molecule is well known to be Pauli forbidden and, therefore, the ground state is the *J* = 1 state.^36,57^ Our calculations also corroborate this selection rule, as projecting onto the *J* = 0 state for all parity and nuclear spin combinations yields an overlap, ***W***^0^, that is in all cases smaller than 10^–6^ atomic units.

We calculated the energy of the first seven states of the molecule, taking into account their proton spin, angular momentum, and parity quantum numbers. As we can only fix  in the Hartree–Fock wave function, we calculated the  expectation value. We sorted the states according to the energy, calculated the energy differences, and compared them to experimental reference values from ref (57). The results presented in Table 3 show that our calculations successfully reproduce the energy ordering when compared to the reference, while also confirming that  is exactly restored. Moreover, although the absolute deviation of the energy differences from the reference increases with rising energy, the relative error remains approximately constant.

**Table 3:** Low-Lying Rotational States of the H_3_^+^ Molecule^a^

*J*		*P*		*E*/Ha	Δ*E*/cm^–1^	Δ*E*_ref_/cm^–1^
1	1/2	–1	0.7500	–1.236663	0	0
1	3/2	1	3.7500	–1.236524	30.59	22.84
2	1/2	1	0.7500	–1.236072	129.71	105.17
2	1/2	–1	0.7500	–1.235652	221.89	173.23
3	3/2	–1	3.7500	–1.235256	308.77	251.22
3	1/2	–1	3.7499	–1.235255	309.24	251.22
3	1/2	1	0.7500	–1.234554	462.87	363.89
3	1/2	–1	0.75			437.90

a The  quantum number is exactly specified in the Hartree–Fock wave function, the quantum numbers *J* and *p* are restored by our projection procedure, while  is not guaranteed to be conserved and is, therefore, evaluated as an expectation value. We rely on the *cc*-pVTZ-mc basis set for the electrons and the PB4-D basis set for the protons, and we applied the Lebedev-trapezoidal rule with 13,020 quadrature points. The reference energies, Δ*E*_ref_, are taken from ref (57).

The results in Table 3 indicate that we have obtained two states with identical energy. Specifically, when targeting the *J* = 3, *p* = −1 state, we set  and , which both resulted in the same energy and  expectation value. Consequently, for both  values, the projection leads to the same state. In those cases where only one  state exists within the subspace defined by specific *J* and *p* quantum numbers, the projection automatically targets the desired  state. However, if multiple  values are possible for a (*J*, *p*) pair, we cannot target a specific  state because only the  quantum number can be specified in the Hartree–Fock ansatz. To target the highest energy *J* = 3 state where , which is the last line of Table 3, we would require an additional projection onto eigenstates of the spin operator.

Finally, since our approach is not a cheap method, we report the timings of the H_3_^+^ projection to address its computational demands: the numerical projection carried out on 64 cores on an Intel(R) Xeon(R) Gold 6136 central processing unit required 1 h and 15 min. Hence, in practice, the timings are modest on modern computing nodes because of the very efficient parallelization. Moreover, for the first approach with nonexponential scaling to target specific rotational states without requiring any prior knowledge of the system, this appears acceptable. We also note that the number of single-point calculations required for ro-vibrational structure calculations based on potential energy surfaces is similar, if not higher, compared to the number of quadrature points in our approach.

## 6 Conclusions

We presented a method for projecting the nuclear-electronic Hartree–Fock wave function onto states with specific rotational and parity quantum numbers. This allows for removing contamination arising from rotationally excited states and, consequently, for obtaining a better variational estimate of the ground-state energy. In contrast to the approximate scheme introduced in ref (32), our method eliminates rotational contributions exactly, thereby addressing the limitations that were highlighted by Sutcliffe.^34^ Furthermore, our ansatz fulfills the requirements recently proposed by Sutcliffe,^58^ allowing for the exact treatment of permutational and rotational symmetry of nuclei, which is lacking in other orbital-based pre-Born–Oppenheimer methods. We demonstrated the reliability of our approach for the prototypical molecules H_2_ and H_3_^+^ by comparing our results to experimental data and the results obtained by Nakai.^12^

This work lays the foundation for further improvements of the NE-HF and related methods. First, a variation-after-projection approach that variationally optimizes the Hartree–Fock wave function in the presence of the projection operator would allow for including orbital relaxation effects and, therefore, further improve the accuracy of the NE-HF energy.^47^ Although correlation effects can be considered by post-Hartree–Fock approaches,^13,18,20,21,24^ this will significantly increase the computational costs. A good balance between costs and accuracy can be achieved within nuclear-electronic density functional theory as demonstrated by Hammes-Schiffer and co-workers^28−31^ to which our approach developed here can be directly applied. Finally, our method can be extended to molecules containing heavy atoms beyond H since the computational scaling of  in the number of orbitals is not prohibitive. Moreover, parallelization is highly efficient. However, to this end, atom-centered nuclear basis functions must be optimized for heavier nuclei based, e.g., on the algorithm described in ref (53).

## Dedication

This work was dedicated to Professor Roland Lindh on the occasion of his 65th birthday.
